# Supplementary material for: HIVprotI: an integrated web based platform for prediction and design of HIV proteins inhibitors
Source: J Cheminform. 2018 Mar 9;10:12. doi: 10.1186/s13321-018-0266-y (PMC5845081; doi:10.1186/s13321-018-0266-y)
Supplement: Supplementary file 2 — Additional file 2. Source code of HIVProtI web server. [file 13321_2018_266_MOESM2_ESM.zip › HIVprotI_Source-code/hivprotI/weka-3-6-6/doc/weka/core/converters/Loader.html]

Loader


---


|  |  |  |
| --- | --- | --- |
| **Overview** \| **Package** \| **Class** \| **Tree** \| **Deprecated** \| **Index** \| **Help** \| **Weka's home** | |  |
| **PREV CLASS**   **NEXT CLASS** | **FRAMES**    **NO FRAMES**     **All Classes** |  |
| SUMMARY: NESTED \| FIELD \| CONSTR \| METHOD | DETAIL: FIELD \| CONSTR \| METHOD |  |


---


## weka.core.converters Interface Loader

**All Superinterfaces:**: RevisionHandler, java.io.Serializable

**All Known Implementing Classes:**: AbstractFileLoader, AbstractLoader, ArffLoader, C45Loader, CSVLoader, DatabaseLoader, LibSVMLoader, SerializedInstancesLoader, SVMLightLoader, TextDirectoryLoader, XRFFLoader

---

``` public interface Loader extends java.io.Serializable, RevisionHandler ```

Interface to something that can load Instances from an input source in some
format.

**Version:**
:   $Revision: 7454 $

**Author:**
:   Mark Hall (mhall@cs.waikato.ac.nz)

---

| **Field Summary** | |
| --- | --- |
| `static int` | `BATCH` |
| `static int` | `INCREMENTAL` |
| `static int` | `NONE`             The retrieval modes |


| **Method Summary** | |
| --- | --- |
| `Instances` | `getDataSet()`             Return the full data set. |
| `Instance` | `getNextInstance(Instances structure)`             Read the data set incrementally---get the next instance in the data set or returns null if there are no more instances to get. |
| `Instances` | `getStructure()`             Determines and returns (if possible) the structure (internally the header) of the data set as an empty set of instances. |
| `void` | `reset()`             Resets the Loader object ready to begin loading. |
| `void` | `setRetrieval(int mode)`             Sets the retrieval mode. |
| `void` | `setSource(java.io.File file)`             Resets the Loader object and sets the source of the data set to be the supplied File object. |
| `void` | `setSource(java.io.InputStream input)`             Resets the Loader object and sets the source of the data set to be the supplied InputStream. |

**Methods inherited from interface weka.core.RevisionHandler**

`getRevision`

**Field Detail**

### NONE

```
static final int NONE
```

:   The retrieval modes

    **See Also:**: Constant Field Values

---


### BATCH

```
static final int BATCH
```

**See Also:**: Constant Field Values

---


### INCREMENTAL

```
static final int INCREMENTAL
```

**See Also:**: Constant Field Values


**Method Detail**

### setRetrieval

```
void setRetrieval(int mode)
```

:   Sets the retrieval mode. Note: Some loaders may not be
    able to implement incremental loading.

    :   **Parameters:**: `mode` - the retrieval mode

---


### reset

```
void reset()
           throws java.lang.Exception
```

:   Resets the Loader object ready to begin loading.
    If there is an existing source, implementations should
    attempt to reset in such a fashion as to be able to
    load from the beginning of the source.

    :   **Throws:**: `java.lang.Exception` - if Loader can't be reset for some reason.

---


### setSource

```
void setSource(java.io.File file)
               throws java.io.IOException
```

:   Resets the Loader object and sets the source of the data set to be
    the supplied File object.

    :   **Parameters:**: `file` - the File **Throws:**: `java.io.IOException` - if an error occurs support loading from a File. ``` public_normal_behavior requires: file != null && (* file exists *); modifiable: model_sourceSupplied, model_structureDetermined; ensures: model_sourceSupplied == true && model_structureDetermined == false; also public_exceptional_behavior requires: file == null || (* file does not exist *); signals: (IOException); ```

---


### setSource

```
void setSource(java.io.InputStream input)
               throws java.io.IOException
```

:   Resets the Loader object and sets the source of the data set to be
    the supplied InputStream.

    :   **Parameters:**: `input` - the source InputStream **Throws:**: `java.io.IOException` - if this Loader doesn't support loading from a File.

---


### getStructure

```
Instances getStructure()
                       throws java.io.IOException
```

:   Determines and returns (if possible) the structure (internally the
    header) of the data set as an empty set of instances.

    :   **Returns:**: the structure of the data set as an empty set of Instances **Throws:**: `java.io.IOException` - if there is no source or parsing fails ``` public_normal_behavior requires: model_sourceSupplied == true && model_structureDetermined == false && (* successful parse *); modifiable: model_structureDetermined; ensures: \result != null && \result.numInstances() == 0 && model_structureDetermined == true; also public_exceptional_behavior requires: model_sourceSupplied == false || (* unsuccessful parse *); signals: (IOException); ```

---


### getDataSet

```
Instances getDataSet()
                     throws java.io.IOException
```

:   Return the full data set. If the structure hasn't yet been determined
    by a call to getStructure then the method should do so before processing
    the rest of the data set.

    :   **Returns:**: the full data set as an Instances object **Throws:**: `java.io.IOException` - if there is an error during parsing or if getNextInstance has been called on this source (either incremental or batch loading can be used, not both). ``` public_normal_behavior requires: model_sourceSupplied == true && (* successful parse *); modifiable: model_structureDetermined; ensures: \result != null && \result.numInstances() >= 0 && model_structureDetermined == true; also public_exceptional_behavior requires: model_sourceSupplied == false || (* unsuccessful parse *); signals: (IOException); ```

---


### getNextInstance

```
Instance getNextInstance(Instances structure)
                         throws java.io.IOException
```

:   Read the data set incrementally---get the next instance in the data
    set or returns null if there are no
    more instances to get. If the structure hasn't yet been
    determined by a call to getStructure then method should do so before
    returning the next instance in the data set.
    If it is not possible to read the data set incrementally (ie. in cases
    where the data set structure cannot be fully established before all
    instances have been seen) then an exception should be thrown.

    :   **Parameters:**: `structure` - the dataset header information, will get updated in case of string or relational attributes **Returns:**: the next instance in the data set as an Instance object or null if there are no more instances to be read **Throws:**: `java.io.IOException` - if there is an error during parsing or if getDataSet has been called on this source (either incremental or batch loading can be used, not both).


---


|  |  |  |
| --- | --- | --- |
| **Overview** \| **Package** \| **Class** \| **Tree** \| **Deprecated** \| **Index** \| **Help** \| **Weka's home** | |  |
| **PREV CLASS**   **NEXT CLASS** | **FRAMES**    **NO FRAMES**     **All Classes** |  |
| SUMMARY: NESTED \| FIELD \| CONSTR \| METHOD | DETAIL: FIELD \| CONSTR \| METHOD |  |


---
